# Genetic Variants at 12p11 and 12q24 Are Associated with Breast Cancer Risk in a Chinese Population

**DOI:** 10.1371/journal.pone.0066519

**Published:** 2013-06-12

**Authors:** Zhenzhen Qin, Yanru Wang, Songyu Cao, Yisha He, Hongxia Ma, Guangfu Jin, Zhibin Hu, Xiaoxiang Guan, Hongbing Shen

**Affiliations:** 1 State Key Laboratory of Reproductive Medicine, Institute of Toxicology, Nanjing Medical University, Nanjing, China; 2 MOE Key Laboratory of Modern Toxicology, Department of Epidemiology and Biostatistics, School of Public Health, Nanjing Medical University, Nanjing, China; 3 Section of Clinical Epidemiology, Jiangsu Key Lab of Cancer Biomarkers, Prevention and Treatment, Cancer Center, Nanjing Medical University, Nanjing, China; 4 Department of Medical Oncology, Jinling Hospital, Southern Medical University, Nanjing, China; Chinese Academy of Medical Sciences, China

## Abstract

**Background:**

A recent genome-wide association study (GWAS) has identified three new breast cancer susceptibility loci at 12p11, 12q24 and 21q21 in populations of European descent. However, because of the genetic heterogeneity, it is largely unknown for the role of these loci in the breast cancer susceptibility in the populations of non-European descent.

**Methodology/Principal Findings:**

Here, we genotyped three variants (rs10771399 at 12p11, rs1292011 at 12q24 and rs2823093 at 21q21) in an independent case–control study with a total of 1792 breast cancer cases and 1867 cancer-free controls in a Chinese population. We found that rs10771399 and rs1292011 were significantly associated with risk of breast cancer with per-allele odds ratios (ORs) of 0.85 (95% confidence interval (CI): 0.76–0.96; *P* = 0.010) and 0.84 (95% CI: 0.76–0.95; *P* = 4.50×10^−3^), respectively, which was consistent with those reported in populations of European descent. Similar effects were observed between ER/PR positive and negative breast cancer for both loci. However, we did not found significant association between rs2823093 and breast cancer risk (OR = 0.97, 95%CI = 0.76–1.24; *P*  = 0.795).

**Conclusions/Significance:**

Our results indicate that genetic variants at 12p11 and 12q24 may also play an important role in breast cancer development in Chinese women.

## Introduction

Breast cancer is the most frequently diagnosed cancer and the leading cause of cancer death among women around the world, accounting for 23% of the new cancer cases and 14% of the cancer deaths in 2008 [1]. In the past 10 years, breast cancer incidence has increased by 20∼30% in China’s urban registries [2]. It has been implicated that menstrual and reproductive factors are associated with risk of breast cancer among both pre- and post-menopausal women in China [3]. Except for environmental exposures including lifestyle and behavioral factors, understanding genetic factors related to breast cancer is also important because identifying such factors may be useful for risk prediction. Over the last 5 years, as a powerful method to investigate the genetic determinants of complex diseases, genome-wide association studies (GWAS) have successfully identified more than 20 common susceptibility loci of breast cancer [4]–[15]. However, most GWAS were conducted in populations of European descent, therefore, it is very necessary to evaluate the generalizability of the GWAS findings in diverse populations across different descents.

Recently, Maya *et al.* carried out a large-scale genome-wide association analysis of breast cancer and further evaluated 72 promising associations in ∼70 000 cases and ∼68 000 controls from 41 case-control studies and 9 breast cancer GWAS. Three new breast cancer susceptibility loci, rs10771399 (12p11), rs1292011 (12q24) and rs2823093 (21q21), were identified in European women, and rs10771399 was also associated with breast cancer risk in Asian women [16]. Following these findings, Antonis *et al.* genotyped rs10771399 and rs1292011 in 12 599 *BRCA1* and 7132 *BRCA2* mutation carriers, and found that rs10771399 near *PTHLH* was also associated with breast cancer risk for *BRCA1* mutation carriers [17]. To evaluate whether these three new identified variants are also associated with breast cancer risk in Chinese women, we conducted a case–control study with 1792 breast cancer cases and 1867 controls in a Chinese population in Jiangsu province of eastern China.

## Materials and Methods

### Ethics Statement

This study was approved by the institutional review board of Nanjing Medical University. The design and performance of current study involving human subjects were clearly described in a research protocol. All participants were voluntary and would complete the informed consent in written before taking part in this research.

### Study subjects

A total of 1792 breast cancer cases and 1867 cancer-free controls were included in the current study, which has been described previously [18]. In brief, all of the breast cancer cases were consecutively recruited, without restrictions of age or histological type, from the First Affiliated Hospital of Nanjing Medical University, the Cancer Hospital of Jiangsu Province and the Gulou Hospital, Nanjing, China, from Jan 2004 to April 2010. These breast cancer patients were newly diagnosed and histopathologically confirmed. Those who had a history of cancer, metastasized cancer from other organs, radiotherapy or chemotherapy were excluded from the case group. All of the cancer-free women controls were randomly selected from more than 30 000 participants in a community-based screening program conducted in Jiangsu province, China. These controls were frequency-matched to the cases on age (5-year interval) and residential area (urban or rural) and collected at the same period as the patients were recruited. All of the cases and the controls were genetically unrelated, ethnic Han Chinese women. After the informed consent was obtained from each participant, a structure questionnaire was completed face-to-face by trained interviewers to collect individual information including demographic data, menstrual and reproductive history, environmental exposure, history of benign breast disease and family history of breast cancer in first-degree relatives (parents, siblings, and children). After the interview, approximately 5-ml of venous blood was collected from each subject. The estrogen receptor (ER) and progesterone receptor (PR) status determined by immunohistochemistry examinations of breast tumors were obtained from the medical records of patients in the hospitals.

### Genotyping

Genotyping was performed using the TaqMan allelic discrimination assay on the platform of 7900HT Real-time PCR System (Applied Biosystems, Foster City, CA) without knowing the subjects’ status (case or control). Two negative controls included in each 384-well reaction plate were used for quality control and the genotyping results were determined by using SDS 2.3 Allelic Discrimination Software (Applied Biosystems).

### Statistical analyses

Hardy–Weinberg equilibrium for the distribution of each single nucleotide polymorphism (SNP) was evaluated using the goodness-of-fit χ^2^ test by comparing the observed genotype frequencies with the expected ones among the controls. Differences between the cases and the controls on the demographic characteristics, selected variables and frequencies of the genotypes were analyzed by using the Student’s t test (for continuous variables) and χ^2^ test (for categorical variables). Logistic regression analyses were employed to evaluate the associations between SNPs and the risk of breast cancer and to estimate the odds ratios (ORs) and their 95% confidence intervals (CIs) with adjustment for age, age at menarche and menopausal status. The heterogeneity of associations between subgroups was assessed using the χ^2^-based Q-test. All of the statistical analyses were performed with Statistical Analysis System software (9.1.3; SAS Institute, Cary, NC, USA).

## Results

Characteristics of the 1792 breast cancer cases and the 1867 cancer-free controls are presented in Table 1. The age between cases and controls was comparable (*P*>0.05). The breast cancer cases showed an earlier age at menarche (*P*<0.05), a later age at first live birth (*P*<0.05) and different distribution of menopausal status (*P*<0.05) when compared with the controls. Among the 1,792 breast cancer subjects, 803 (55.5%) cases were ER positive while 810 (56.1%) cases were PR positive.

**Table 1:** Distributions of select variables between breast cancer cases and cancer-free controls.

Variables	Cases (N = 1792)	Controls (N = 1867)	*P* value
Age, years (mean±s.d.)	50.69±11.38	50.01±12.07	0.077
Age at menarche, years (mean±s.d.)^a^	15.21±1.90	16.05±1.86	<0.001
Age at first live birth, years (mean±s.d.)^b^	25.60±3.32	24.51±3.03	<0.001
Age at natural menopause, years (mean±s.d.)^c^	49.78±3.45	49.42±3.84	0.051
Menopausal status			<0.001
Premenopausal	850	967	
Natural menopausal	765	850	
Unnatural menopausal	142	25	
Estrogen receptor (ER)			
Positive	803		
Negative	643		
Progesterone receptor (PR)			
Positive	810		
Negative	634		

a Age at menarche information was available in 1755 breast cancer cases and 1858 controls;

b Age at first live birth information was available in 1680 breast cancer cases and 1815 controls; 65 subjects were nulliparous while 99 were with missing information;

c Age at natural menopause information was available in 743 breast cancer cases and 809 controls.

The genotype distributions of the 3 SNPs in cases and controls and their associations with breast cancer risk were summarized in Table 2. The observed genotype frequencies of 3 SNPs followed Hardy–Weinberg equilibrium in the controls (*P*>0.05 for all SNPs). Significant associations were observed between rs10771399 at 12p11 and rs1292011 at 12q24 and breast cancer risk (*P* = 0.010 and 4.50×10^−3^, respectively). The G allele of rs10771399 and the G allele of rs1292011 were associated with a decreased risk of breast cancer (per-allele OR = 0.85, 95% CI: 0.76–0.96, *P* = 0.010; and per-allele OR = 0.84, 95% CI: 0.76–0.95, *P* = 4.50×10^−3^, respectively), which are consistent with those reported by Maya et al.[16]. However, no significant association was observed between rs2823093 at 21q21 and breast cancer risk.

**Table 2:** The associations of three loci at 12p11, 12q24 and 21q21 with breast cancer risk.

Location	Locus	MAF a	Cases (N = 1792)		Controls (N = 1867)		OR(95%CI) b		*P* b
			N	%	N	%			
12p11	rs10771399 A>G	0.258/0.125							
	AA		1145	65.69	1145	62.23	1.00		
	AG		536	30.75	602	32.72	0.89(0.77–1.04)		0.146
	GG		62	3.56	93	5.05	0.63(0.44–0.89)		9.24×10^−3^
	Per allele c						0.85(0.76–0.96)		0.010
12q24	rs1292011 A>G	0.233/0.392							
	AA		1080	61.36	1036	56.27	1.00		
	AG		598	33.98	695	37.75	0.83(0.72–0.96)		0.013
	GG		82	4.66	110	5.98	0.75(0.55–1.02)		0.064
	Per allele c						0.84(0.76–0.95)		4.50×10^−3^
21q21	rs2823093 G>A	0.042/0.300							
	GG		1645	92.52	1683	92.22	1.00		
	AG		130	7.31	137	7.51	0.97(0.75–1.26)		0.843
	AA		3	0.17	5	0.27	0.83(0.18–3.90)		0.817
	Per allele c						0.97(0.76–1.24)		0.795

a Minor allele frequency between Asian/European population from 1000 Genomes project;

b Adjusted for age, age at menarche and menopausal status;

c Derived from additive model.

We further evaluated the associations of rs10771399 and rs1292011 on breast cancer risk in subgroups stratified by age, age at menarche and first live birth, and menopausal status (premenopausal and natural menopausal). As shown in Table 3, there was no significant difference between subgroups for associations of rs10771399 and rs1292011 with breast cancer risk (*P* for heterogeneity >0.05). Similar per-allele ORs were observed between ER/PR positive and negative breast cancer for both SNPs.

**Table 3:** Stratified analysis on the associations of rs10771399 at 12p11 and rs1292011 at 12q24 with breast cancer risk.

Characteristics	rs10771399				rs1292011			
	Case	Control	OR(95%CI) a	*P* b	Case	Control	OR(95%CI)^a^	*P* b
Age								
<50	573/267/27	622/306/51	0.89(0.75–1.05)	0.375	515/317/44	547/370/61	0.91(0.78–1.07)	0.185
≥50	572/269/35	523/296/42	0.80(0.67–0.95)		565/281/38	489/325/49	0.78(0.66–0.92)	
Age at menarche								
<15	443/197/32	237/126/25	0.84(0.68–1.03)	0.927	408/243/25	234/129/26	0.92(0.74–1.14)	0.374
≥15	679/328/27	905/472/66	0.85(0.73–0.98)		652/339/57	797/562/84	0.82(0.72–0.94)	
Age at first live birth								
<25	389/179/26	572/293/46	0.90(0.75–1.09)	0.471	372/200/31	532/332/47	0.91(0.76–1.08)	0.162
≥25	682/328/29	544/289/45	0.82(0.69–0.97)		649/352/47	476/342/61	0.77(0.66–0.90)	
Menopausal status								
Premenopausal	548/255/25	592/313/53	0.80(0.68–0.95)	0.497	503/290/42	541/353/65	0.89(0.76–1.04)	0.242
Postmenopausal^c^	490/227/28	521/274/37	0.88(0.73–1.05)		480/241/31	465/323/44	0.77(0.65–0.92)	
ER status								
Positive	505/247/27		0.86(0.74–1.01)	0.992	491/259/36		0.82(0.71–0.95)	0.787
Negative	408/201/21		0.86(0.73–1.02)		390/216/29		0.85(0.72–0.99)	
PR status								
Positive	518/248/25		0.84(0.72–0.98)	0.709	504/253/38		0.80(0.70–0.94)	0.574
Negative	397/196/23		0.88(0.74–1.04)		377/220/27		0.86(0.73–1.01)	

a Adjusted for age, age at menarche, menopausal status where appropriate;

b *P* for heterogeneity;

c Postmenopausal status for natural menopause.

## Discussion

In this study, we evaluated the associations of genetic variants at 12p11 (rs10771399), 12q24 (rs1292011) and 21q21 (rs2823093) with breast cancer susceptibility in an independent case–control study with 1792 breast cancer cases and 1867 controls in a Chinese population. We found that rs10771399 and rs1292011 but not rs2823093 were significantly associated with altered risk of breast cancer in our population. The OR of 0.85 for rs10771399 in this Chinese population was similar to those observed in European populations initially reported by Maya *et al.* (OR = 0.85) [16] and subsequently confirmed by Antonis *et al.* (OR = 0.87) [17]. Of note, we detected a more protective effect of rs10771399 in our population (OR = 0.85) than that reported by Maya *et al.* (OR = 0.92). These findings provided new evidence for the usefulness of the breast cancer susceptibility loci at 12p11 and 12q24 in multiple populations with various descents.

Since rs10771399 was a proxy of 12p11 region, we further functionally annotated the linkage disequilibrium (LD) block containing rs10771399 using the UCSC genome browser, and found that none of known genes overlapped with this region (Figure S1). The nearest gene *PTHLH* encodes a protein that regulates endochondral bone development and epithelial-mesenchymal interactions during the formation of the mammary glands, whose receptor is responsible for most cases of humoral hypercalcemia of malignancy [19]. This gene had been approved to be involved in the metastasis of breast cancer [20]. We searched SNPs in strong LD (r^2^>0.8) with rs10771399 based on CHB/JPT data of 1000 Genome pilot (Table S1), and predicted the biological functions of these variants using an online tool, RegulomeDB [21]. As shown in Table S1, three SNPs (rs788463, rs10843066 and rs7957915) were predicted to be in regulatory elements and likely to affect binding of transcriptional factors. For example, rs788463 is in 2.4 kb upstream of the lead SNP rs10771399 and in complete LD (r^2^ = 1.0) with rs10771399. Analyses of DNase Footprinting and Position-Weight Matrix (PWM) suggest that rs788463 is in the binding sites of transcriptional factors Osf2, C/EBP and C/EBPalpha. ChIP-seq data suggests this variant located at sites of multiple histone modification marks, including H3k27me3, H3k4me1 and H3k4me2. Taken together, these evidences indicate that genetic variants at 12p11 (such as rs788463) may affect the binding sites of transcriptional factors (such as C/EBP), modify the function of regulatory elements and finally involve in the development of breast cancer. However, the potential target genes are unclear and the above statements also need to be experimentally validated in the future.

Similarly, we also functionally annotated the LD block containing rs1292011 at 12q24 (Figure S2) and performed functional prediction for those SNPs in strong LD with rs1292011 (r^2^>0.8) (Table S2). There are no genes in the LD region of rs1292011. The variant rs1391721, which is close to lead SNP rs1292011 (390 bp upstream) and highly correlated with it (r^2^ = 0.96), implies a functional potential in effecting the combination between transcription factors and their binding site. This variant falls on a position within a footprint containing an Evi-1 and GATA-1/2/3 motifs in MCF-7 cell line and substantially affect footprinting of these motifs, resulting in allelic imbalance in chromatin accessibility. Again, these evidences are kind of bioinformatics prediction and functional experiments are warranted to validate these insights.

We did not observe a significant evidence of association between rs2823093 and breast cancer risk in our study. In the study of Maya *et al.* [16], they also reported a negative association of rs2823093 in women of Asian ancestry (OR = 1.14, 95%CI = 0.93–1.40) from BCAC (Breast Cancer Association Consortium). Such discrepancy is common between different populations. For example, with a consortium effort including 23637 breast cancer patients and 25579 controls of East Asian ancestry, Zheng *et al.* [22] investigated 67 independent susceptibility loci of breast cancer identified by GWAS primarily in European-ancestry populations and found 31 loci at *P*<0.05 in a direction consistent with that reported previously. In terms of the inconsistence of rs2823093 in this study, one of the explanations is the genetic heterogeneity between populations that the identified SNP rs2823093 may be a good proxy of causal variants at 21q21 in population of European descent but poor in Asians. Moreover, as shown in Table 2, in contrast to the other two replicated SNPs, the minor allele frequency of rs2823093 is lower in Asian population (MAF =  0.042) than those in population of European descent (MAF =  0.300), which also reflects the ethnical difference and may result in a low statistical power in this study. For another explanation, the locus of 21q21 may be out of effect in Asian women and inactive in the absence of a specific exposure in Asians, which, however, is common to European women. Taken together, discrepancy between different ethnicities for association between genetic variants and breast cancer risk could be explained partly by the different genetic background. Other possible explanations also include disease heterogeneity, study design and sample size. Well-designed fine-mapping studies with large sample size following resequencing this region may help to clearly evaluate the role of 21q21 in breast cancer development in populations of non-European descent.

In summary, our study evaluated three new breast cancer loci in a Chinese population that were identified in populations of European descent. Our results suggest that genetic variants at 12p11 and 12q24, tagged by rs10771399 and rs1292011, respectively, may also play an important role in the susceptibility of breast cancer in Chinese women. Further studies are warranted to clarify the biological mechanisms of these two loci with breast cancer risk and determine the causal variants in breast carcinogenesis.

## Supporting Information

Figure S1 **Overview of the LD block containing rs10771399 at 12p11 from the UCSC browser (NCBI36/hg18).** A 250-kb window within upstream and downstream of the proxy SNP rs10771399 at 12p11 was annotated. Linkage disequilibrium (LD) region was generated using the HaploView 4.2 software according to HapMap II+III CHB data.
(DOC)

Figure S2 **Overview of the LD block containing rs1292011 at 12q24 from the UCSC browser (NCBI36/hg18).** A 250-kb window within upstream and downstream of the proxy SNP rs1292011 at 12q24 was annotated. Linkage disequilibrium (LD) region was generated using the HaploView 4.2 software according to HapMap II+III CHB data.
(DOC)

Table S1 **SNPs in strong LD (r^2^>0.8) with rs10771399 at 12p11 based on 1000 Genome pilot CHB/JPT data and biological function scores predicted by RegulomeDB.**
(DOC)

Table S2 **SNPs in strong LD (r2>0.8) with rs1292011 at 12q24 based on 1000 Genome pilot CHB/JPT data and biological function scores predicted by RegulomeDB.**
(DOC)

## References

[1] Jemal A, Bray F (2011) Center MM, Ferlay J, Ward E, et al (2011) Global cancer statistics. CA Cancer J Clin 61: 69–90. 21296855 10.3322/caac.20107

[2] Porter P (2008) "Westernizing" women's risks? Breast cancer in lower-income countries. N Engl J Med 358: 213–216. 18199859 10.1056/NEJMp0708307

[3] Long N, Moore MA, Chen W, Gao CM, Lai MS, et al (2010) Cancer epidemiology and control in north-East Asia - past, present and future. Asian Pac J Cancer Prev 11 Suppl 2107–148. 20553072

[4] Easton DF, Pooley KA, Dunning AM, Pharoah PD, Thompson D, et al (2007) Genome-wide association study identifies novel breast cancer susceptibility loci. Nature 447: 1087–1093. 17529967 10.1038/nature05887 PMC2714974

[5] Hunter DJ, Kraft P, Jacobs KB, Cox DG, Yeager M, et al (2007) A genome-wide association study identifies alleles in FGFR2 associated with risk of sporadic postmenopausal breast cancer. Nat Genet 39: 870–874. 17529973 10.1038/ng2075 PMC3493132

[6] Stacey SN, Manolescu A, Sulem P, Rafnar T, Gudmundsson J, et al (2007) Common variants on chromosomes 2q35 and 16q12 confer susceptibility to estrogen receptor-positive breast cancer. Nat Genet 39: 865–869. 17529974 10.1038/ng2064

[7] Gold B, Kirchhoff T, Stefanov S, Lautenberger J, Viale A, et al (2008) Genome-wide association study provides evidence for a breast cancer risk locus at 6q22.33. Proc Natl Acad Sci U S A 105: 4340–4345. 18326623 10.1073/pnas.0800441105 PMC2393811

[8] Stacey SN, Manolescu A, Sulem P, Thorlacius S, Gudjonsson SA, et al (2008) Common variants on chromosome 5p12 confer susceptibility to estrogen receptor-positive breast cancer. Nat Genet 40: 703–706. 18438407 10.1038/ng.131

[9] Zheng W, Long J, Gao YT, Li C, Zheng Y, et al (2009) Genome-wide association study identifies a new breast cancer susceptibility locus at 6q25.1. Nat Genet 41: 324–328. 19219042 10.1038/ng.318 PMC2754845

[10] Ahmed S, Thomas G, Ghoussaini M, Healey CS, Humphreys MK, et al (2009) Newly discovered breast cancer susceptibility loci on 3p24 and 17q23.2. Nat Genet 41: 585–590. 19330027 10.1038/ng.354 PMC2748125

[11] Thomas G, Jacobs KB, Kraft P, Yeager M, Wacholder S, et al (2009) A multistage genome-wide association study in breast cancer identifies two new risk alleles at 1p11.2 and 14q24.1 (RAD51L1). Nat Genet 41: 579–584. 19330030 10.1038/ng.353 PMC2928646

[12] Turnbull C, Ahmed S, Morrison J, Pernet D, Renwick A, et al (2010) Genome-wide association study identifies five new breast cancer susceptibility loci. Nat Genet 42: 504–507. 20453838 10.1038/ng.586 PMC3632836

[13] Long J, Cai Q, Shu XO, Qu S, Li C, et al (2010) Identification of a functional genetic variant at 16q12.1 for breast cancer risk: results from the Asia Breast Cancer Consortium. PLoS Genet 6: e1001002. 20585626 10.1371/journal.pgen.1001002 PMC2891809

[14] Fletcher O, Johnson N, Orr N, Hosking FJ, Gibson LJ, et al (2011) Novel breast cancer susceptibility locus at 9q31.2: results of a genome-wide association study. J Natl Cancer Inst 103: 425–435. 21263130 10.1093/jnci/djq563

[15] Cai Q, Long J, Lu W, Qu S, Wen W, et al (2011) Genome-wide association study identifies breast cancer risk variant at 10q21.2: results from the Asia Breast Cancer Consortium. Hum Mol Genet 20: 4991–4999. 21908515 10.1093/hmg/ddr405 PMC3221542

[16] Ghoussaini M, Fletcher O, Michailidou K, Turnbull C, Schmidt MK, et al (2012) Genome-wide association analysis identifies three new breast cancer susceptibility loci. Nat Genet 44: 312–318. 22267197 10.1038/ng.1049 PMC3653403

[17] Antoniou AC, Kuchenbaecker KB, Soucy P, Beesley J, Chen X, et al (2012) Common variants at 12p11, 12q24, 9p21, 9q31.2 and in ZNF365 are associated with breast cancer risk for BRCA1 and/or BRCA2 mutation carriers. Breast Cancer Res 14: R33. 22348646 10.1186/bcr3121 PMC3496151

[18] Chen J, Jiang Y, Liu X, Qin Z, Dai J, et al (2012) Genetic variants at chromosome 9p21, 10p15 and 10q22 and breast cancer susceptibility in a Chinese population. Breast Cancer Res Treat 132: 741–746. 22198471 10.1007/s10549-011-1927-y

[19] Suva LJ, Winslow GA, Wettenhall RE, Hammonds RG, Moseley JM, et al (1987) A parathyroid hormone-related protein implicated in malignant hypercalcemia: cloning and expression. Science 237: 893–896. 3616618 10.1126/science.3616618

[20] Kakonen SM, Mundy GR (2003) Mechanisms of osteolytic bone metastases in breast carcinoma. Cancer 97: 834–839. 12548583 10.1002/cncr.11132

[21] Boyle AP, Hong EL, Hariharan M, Cheng Y, Schaub MA, et al (2012) Annotation of functional variation in personal genomes using RegulomeDB. Genome Res 22: 1790–1797. 22955989 10.1101/gr.137323.112 PMC3431494

[22] Zheng W, Zhang B, Cai Q, Sung H, Michailidou K,,et al..(2013) Common genetic determinants of breast-cancer risk in East Asian women: a collaborative study of 23 637 breast cancer cases and 25 579 controls. Hum Mol Genet. Mar 27. [Epub ahead of print]. 10.1093/hmg/ddt089 PMC3658167 23535825

