# Supplementary material for: Genetic Variants at 12p11 and 12q24 Are Associated with Breast Cancer Risk in a Chinese Population
Source: PLoS One. 2013 Jun 12;8(6):e66519. doi: 10.1371/journal.pone.0066519 (PMC3680498; doi:10.1371/journal.pone.0066519)

Figure.S2 Overview of the LD block containing rs1292011 at 12q24 from the UCSC browser (NCBI36/hg18). A 250-kb window within upstream and downstream of the proxy SNP rs1292011 at 12q24 was annotated. Linkage disequilibrium (LD) region was generated using the HaploView 4.2 software according to HapMap II+III CHB data.


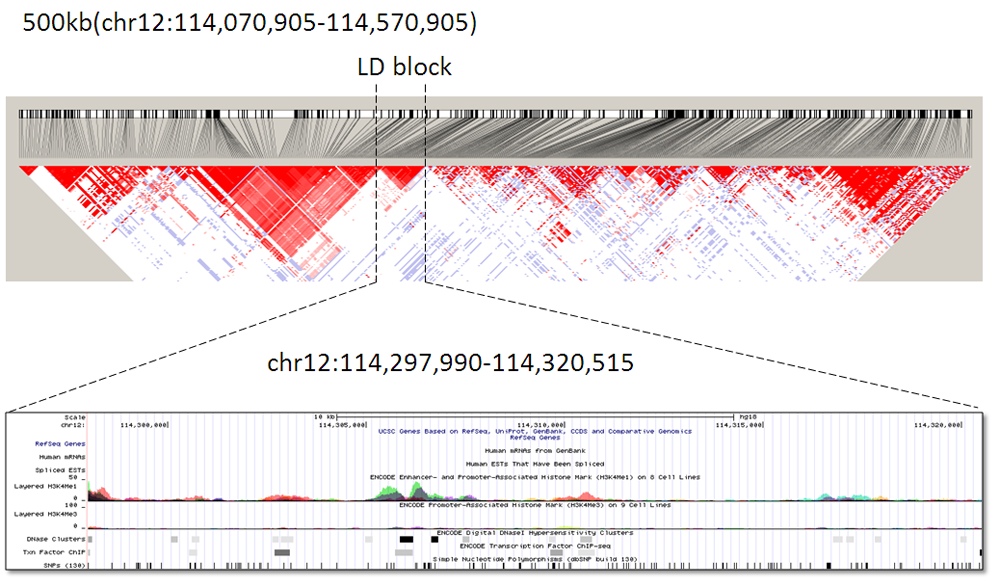

Supplement: Figure S2 — Overview of the LD block containing rs1292011 at 12q24 from the UCSC browser (NCBI36/hg18). A 250-kb window within upstream and downstream of the proxy SNP rs1292011 at 12q24 was annotated. Linkage disequilibrium (LD) region was generated using the HaploView 4.2 software according to HapMap II+III CHB data. (DOC) [file pone.0066519.s002.doc]
