# Supplementary material for: Genetic Variants at 12p11 and 12q24 Are Associated with Breast Cancer Risk in a Chinese Population
Source: PLoS One. 2013 Jun 12;8(6):e66519. doi: 10.1371/journal.pone.0066519 (PMC3680498; doi:10.1371/journal.pone.0066519)
Supplement: Table S1 — SNPs in strong LD (r2>0.8) with rs10771399 at 12p11 based on 1000 Genome pilot CHB/JPT data and biological function scores predicted by RegulomeDB. (DOC) [file pone.0066519.s003.doc]

**Table.S1** SNPs in strong LD (r^2^>0.8) with rs10771399 at 12p11 based on 1000 Genome pilot CHB/JPT data and biological function scores predicted by RegulomeDB.

| SNP | Position (bp) | Distance (bp)^a^ | Major allele | Minor allele | MAF ^b^ | r^2 c^ | Score ^d^ |
| --- | --- | --- | --- | --- | --- | --- | --- |
| rs805511 | 28031000 | 15347 | A | C | 0.25 | 0.957 | 6 |
| rs805510 | 28031113 | 15234 | C | T | 0.225 | 0.834 | 7 |
| rs1314085 | 28031520 | 14827 | G | T | 0.258 | 1 | 7 |
| rs1314084 | 28031544 | 14803 | T | C | 0.258 | 1 | 7 |
| rs1267211 | 28032096 | 14251 | T | C | 0.258 | 1 | 7 |
| rs1267210 | 28032185 | 14162 | C | T | 0.258 | 1 | 5 |
| rs1267208 | 28034217 | 12130 | C | T | 0.258 | 1 | 7 |
| rs251955 | 28034729 | 11618 | T | C | 0.258 | 1 | 7 |
| rs1267206 | 28034979 | 11368 | C | A | 0.258 | 1 | 6 |
| rs1267205 | 28035054 | 11293 | C | T | 0.25 | 0.957 | 7 |
| rs1267204 | 28035245 | 11102 | T | C | 0.25 | 0.957 | 6 |
| rs251954 | 28035738 | 10609 | G | C | 0.267 | 0.958 | 7 |
| rs1270113 | 28036688 | 9659 | C | T | 0.258 | 1 | 7 |
| rs251952 | 28037217 | 9130 | T | C | 0.258 | 1 | 7 |
| rs251951 | 28037709 | 8638 | A | G | 0.258 | 1 | 7 |
| rs251950 | 28037757 | 8590 | A | T | 0.258 | 1 | 7 |
| rs805515 | 28038348 | 7999 | C | T | 0.258 | 1 | 7 |
| rs805513 | 28039908 | 6439 | G | A | 0.258 | 1 | 6 |
| rs788458 | 28040835 | 5512 | C | T | 0.258 | 1 | 7 |
| rs2737456 | 28040879 | 5468 | T | C | 0.258 | 1 | 6 |
| rs2737455 | 28040881 | 5466 | T | A | 0.258 | 1 | 6 |
| rs788459 | 28040926 | 5421 | T | C | 0.258 | 1 | 7 |
| rs788460 | 28041539 | 4808 | A | T | 0.258 | 1 | 7 |
| rs788461 | 28041755 | 4592 | T | C | 0.258 | 1 | 7 |
| rs788462 | 28042285 | 4062 | T | A | 0.258 | 1 | 7 |
| rs813722 | 28043311 | 3036 | A | G | 0.258 | 1 | 7 |
| rs788463 | 28043915 | 2432 | A | T | 0.258 | 1 | 2b |
| rs788464 | 28044285 | 2062 | A | G | 0.25 | 0.957 | 5 |
| rs2737448 | 28044527 | 1820 | T | C | 0.258 | 1 | 6 |
| rs2737447 | 28044794 | 1553 | T | C | 0.258 | 1 | 7 |
| rs2619415 | 28045014 | 1333 | G | T | 0.258 | 1 | 6 |
| rs1838564 | 28046162 | 185 | G | A | 0.258 | 1 | 7 |
| rs10771399 | 28046347 | 0 | A | G | 0.258 | 1 | 7 |
| rs2619411 | 28052423 | 6076 | G | A | 0.25 | 0.957 | 7 |
| rs56003999 | 28053015 | 6668 | T | C | 0.258 | 1 | 7 |
| rs11049276 | 28054098 | 7751 | A | G | 0.258 | 1 | 6 |
| rs11049277 | 28054146 | 7799 | C | G | 0.258 | 1 | 7 |
| rs11049278 | 28054767 | 8420 | T | C | 0.258 | 1 | 7 |
| rs11049279 | 28055030 | 8683 | G | C | 0.258 | 1 | 5 |
| rs11049280 | 28055244 | 8897 | C | T | 0.267 | 0.958 | 6 |
| rs11049281 | 28055653 | 9306 | T | C | 0.258 | 1 | 7 |
| rs11049282 | 28055788 | 9441 | G | T | 0.258 | 1 | 7 |
| rs11049283 | 28055900 | 9553 | A | C | 0.258 | 1 | 6 |
| rs2619433 | 28057324 | 10977 | A | G | 0.258 | 1 | 7 |
| rs2619432 | 28057605 | 11258 | A | G | 0.25 | 0.957 | 7 |
| rs809291 | 28058868 | 12521 | G | A | 0.258 | 1 | 7 |
| rs788455 | 28058886 | 12539 | T | C | 0.258 | 1 | 6 |
| rs76401589 | 28060602 | 14255 | C | T | 0.258 | 1 | 7 |
| rs788457 | 28061356 | 15009 | A | G | 0.25 | 0.957 | 7 |
| rs809816 | 28061441 | 15094 | T | C | 0.258 | 1 | 5 |
| rs11049285 | 28062795 | 16448 | T | A | 0.25 | 0.957 | 7 |
| rs11049286 | 28063014 | 16667 | G | C | 0.258 | 1 | 7 |
| rs10843056 | 28063413 | 17066 | T | C | 0.267 | 0.958 | 4 |
| rs10843057 | 28063609 | 17262 | T | G | 0.258 | 1 | 5 |
| rs10843058 | 28063812 | 17465 | G | A | 0.258 | 1 | 6 |
| rs10843059 | 28064462 | 18115 | G | C | 0.258 | 1 | 7 |
| rs7958748 | 28064939 | 18592 | G | A | 0.258 | 1 | 7 |
| rs10843061 | 28065836 | 19489 | T | C | 0.258 | 1 | 6 |
| rs11049290 | 28065946 | 19599 | A | G | 0.258 | 1 | 4 |
| rs10843062 | 28066307 | 19960 | C | T | 0.258 | 1 | 4 |
| rs10843063 | 28067039 | 20692 | T | A | 0.258 | 1 | 7 |
| rs10843064 | 28067054 | 20707 | G | C | 0.258 | 1 | 7 |
| rs10843065 | 28067848 | 21501 | G | C | 0.258 | 1 | 4 |
| rs10843066 | 28068064 | 21717 | T | C | 0.258 | 1 | 2b |
| rs10843067 | 28068220 | 21873 | T | C | 0.258 | 1 | 6 |
| rs10843068 | 28068448 | 22101 | T | G | 0.242 | 0.915 | 5 |
| rs10843069 | 28068925 | 22578 | C | T | 0.258 | 1 | 6 |
| rs12231591 | 28069422 | 23075 | T | C | 0.258 | 1 | 7 |
| rs12230582 | 28070342 | 23995 | C | T | 0.258 | 1 | 7 |
| rs11049292 | 28070749 | 24402 | A | G | 0.258 | 1 | 5 |
| rs11049293 | 28070968 | 24621 | T | C | 0.25 | 0.957 | 4 |
| rs7957915 | 28071769 | 25422 | A | G | 0.283 | 0.881 | 2b |
| rs57498301 | 28072287 | 25940 | T | C | 0.283 | 0.881 | 4 |
| rs16932559 | 28072500 | 26153 | T | C | 0.283 | 0.881 | 4 |
| rs11049294 | 28072670 | 26323 | G | C | 0.283 | 0.881 | 5 |
| rs11049295 | 28073519 | 27172 | T | C | 0.283 | 0.881 | 7 |

^a^ Physical distance from the proxy SNP rs10771399;

^b^ Minor allele frequency based on CHB/JPT data of 1000 Genome pilot;

^c^ The value for linkage disequilibrium between each SNP and rs10771399;

^d^ The scoring system of RegulomeDB was developed based on functional confidence of a variant. Lower scores indicate increasing evidence for a variant to be located in a functional region. Variants that are known eQTLs for genes, and thus have been shown to be associated with expression, as most likely to be significant are labeled as Category 1. Sub-categories within Category 1 indicate additional annotations from the most confident (1a, which has TF binding, a motif for that TF, and a DNase footprint) to the least confident (1f, which has only TF binding or a DNase peak).Category 2(a–c) demonstrates direct evidence of binding through ChIP-seq and DNase with either a matched PWM to the ChIP-seq factor or a DNase footprint. Category 3(a–b) is considered less confident in affecting binding due to a more incomplete set of evidence. Categories 4–6 lack evidence of the variant actually disrupting the site of binding. These include DNase and ChIP-seq evidence (Category 4), DNase or ChIP-seq evidence (Category 5), or any single annotation not in the above categories (Category 6). 58% of eQTL SNVs which are not associated with any other functional annotation are labeled as Category 6. Category 7 implies variant with no annotation.
